# An Open-Source Relational Network Derivation Script in R for Modeling and Visualizing Complex Behavior for Scientists and Practitioners

**DOI:** 10.3389/fpsyg.2022.914485

**Published:** 2022-06-15

**Authors:** Patrick Smith, Steven C. Hayes

**Affiliations:** Department of Psychology, University of Nevada, Reno, Reno, NV, United States

**Keywords:** relational networks, R, graph networks, relational derivation, relational frame theory

## Abstract

Relational models of cognition provide parsimonious and actionable models of generative behavior witnessed in humans. They also inform many current computational analogs of cognition including Deep Neural Networks, Reinforcement Learning algorithms, Self-Organizing Maps, as well as blended architectures that are outperforming traditional semantic models. The black box nature of these computer models artificially limits scientific and applied progress and human computer interaction. This paper presents a first in the field attempt to model relational processes using logical derivation scripts and network graph visualizations written in the open-source R language. These tools are presented as a way for researchers and practitioners to begin to explore more complex relational models in a manner that can advance the theory and empirical science, as well as prepare the field for future collaborations with advanced computational models of cognition.

## Introduction

Relational models of language and cognition are receiving increased attention in both cognitive science and behavioral psychology. For example, cognitive researchers have shown that relational reasoning is central to fluid intelligence developmentally (Crone et al., 2009); behavioral psychologists have found that training in relational reasoning can impact intelligence in normally developing (May et al., 2022) and developmentally delayed populations (Dixon et al., 2021). Artificial general intelligence (AGI) is similarly drawing together research traditions in their focus on relational reasoning (Johansson, 2019). For example, “relation networks” are being successfully explored in neural network models of AGI (Santoro et al., 2017) and Relational Frame Theory (RFT: Hayes et al., 2001) is being effectively used to address shortfalls of traditional structural semantics models in AGI implementations (Edwards et al., 2022).

In order to explore the broader implications of assessing and training relational reasoning for complex issues in human cognition it is necessary to have both highly specified tasks (e.g., see Cortes et al., 2021) and carefully crafted analytic and descriptive models. We will use the example of RFT to explain why that is so.

Relational frame theory accounts for generativity in human language through a learned behavior of combining two or more previously trained relations into novel derived (not directly trained) relations. For example, if *A* is less than *B* and *B* is less than *C*, by combinatorial derivation *A* must be less than *C*. Combinatorial derivation, along with learning to respond in reverse to a trained relation (i.e., Mutual Entailment: If *A* > *B* then *B* < *A*) generate relational responding behavioral repertoires rooted in human language at the rate of ~ 2^(*n*–1)^ for any given set of *n* trained relations with shared relata.

This massive generativity leads to enormously complex relational networks and systems such as the entire corpus of human written knowledge. The expansive quality of human cognition when it is viewed relationally means, on the one hand, that simplified models and procedures used in controlled research are subject to criticisms of excessive extrapolation. On the other hand, it means that more naturalistic and expansive models that may describe and predict human behavior can either become inexact or entirely incomprehensible when they are examined as a collection of discrete elements.

A variety of computational systems and analogs of relational models have emerged to facilitate research and understanding because of this conundrum. Some of the computational analogs informed by RFT have been drawn from machine learning expert systems and artificial intelligence neural networks. Examples include the use of Deep Neural Networks (DNN) to detect effects in multiple baseline single case design graphed data (Lanovaz and Bailey, 2020) and forecast human participant learning of trigonometry (Ninness et al., 2019; Ninness and Ninness, 2020); the use of Kohonen Self-Organizing Maps (SOM: Kohonen, 1988) for behavioral pattern detection in legislature voting, breast cancer diagnosis (Ninness et al., 2012), visual symmetry detection (Dresp-Langley and Wandeto, 2021), and surgical expertise detection (Dresp-Langley et al., 2021); and blends of DNN and SOM architectures to model decision making in child welfare systems (Ninness et al., 2021). Additional work with Connectionist Models (CM) has provided confirmatory validation of methodological nuances in relational training sequencing for humans (Lyddy and Barnes-Holmes, 2007).

Much of the above work is evaluated solely on predictive validity or goodness of fit to untrained data. Relationally informed computer analogs provide a yes/no decision or predicted value and confidence interval given an input set of data, but otherwise do not provide interpretable output to contextualize the results. This generic criticism of AI systems is called “the black box problem” (Bunge, 1963) and the need to both experimentally analyze these systems and the possibility of human–computer collaboration with them has highlighted the need to generate descriptions of relational networks that are interpretable by humans. One specific conceptual description of such output is a relational network represented as a directional graph of vertices and edges where each vertex represents some relata and edges represent relations. A complex version of this graph representation generated by a blended AI architecture is conceptually described by Edwards et al. (2022). The present paper describes scripts for the R Statistical Software package (R Core Team, 2020) that constitutes a graph network generator and visualizer that conform to a relational learning model. It is provided as a means of exploring how such a visual representation of relational responding might complement applied and experimental practices.

These scripts are designed to intake a base set of relational statements and generate tabular output of all expected derived relations. That output can be interpreted on its own or visualized with the accompanying visualization script. The immediate purpose of the package is to provide a tool for relational cognition researchers to model and visualize the deriving of relationships that is human readable, reliable, quick, and repeatable to a level of complexity not previously available. The following sections of this paper include a detailed description of the specific steps taken to translate theory into code (see section “Materials and Methods”), a tutorial on how to use the code itself with accompanying reference outputs (see section “Results/Tutorial”), and an extensive discussion of the considerations, limitation, and future directions (see section “Discussion”). It is not necessary to be familiar with the theory to start working with this package. A reader who wishes simply to get the code up and running *via* the tutorials can skip to “Results/Tutorial”.

## Materials and Methods

A consistent method of computer automated relational derivation is not currently available. RFT is being used as a base model of doing so because of its clarity and simplicity regarding the minimal requirements of relational learning. Outside of the computer coding itself, one key element required for this kind of automation is a formalized set of steps that can reliably derive relations for both mutual and combinatorial derivation. In this first attempt we set aside issues of contextual control over derived stimulus relations and the separate contextual control of functions that are altered by these relations. Instead, in a first attempt to create just an automation tool, the first author (PS) has focused on formalizing the logical steps of derivation at the mutual and combinatorial levels for three of the most common relations used in RFT research and application, the equivalence relation (SAME or “=”) and the simple comparative relations GREATER THAN (>) and LESS THAN (<).The next sections will attempt to describe each rigorously and following sections will discuss some of the challenges that surfaced and how they were addressed in software package now available for use under a General Public License version 3 (GPLv3) copyleft license, written in the R Statistical language (R Core Team, 2020), and available on GitHub.

### Equivalence Derivation

#### Mutual Entailment

At the level of mutual entailment, equivalence relations are defined by symmetric responding (Sidman et al., 1982; Sidman and Tailby, 1982). That is, for any relational response to Stimulus *A* as it relates to Stimulus *B*, the response to Stimulus *B* as it relates to Stimulus *A* is functionally identical. One of the most common incidences of equivalence responding in human behavior are responding to words as if they are the event we equate them to and vice versa. The symbolic representation of equivalence is commonly the mathematical equals sign (=). If an equivalence relation is trained (e.g., *A* = *B*) then the mutually entailed equivalence response is described by a relational statement that simply swaps the stimuli around the equals sign (e.g., *B* = *A*). Pseudo code for mutual derivation of equivalence relations would look something like the following:

Input: One relational statement composed of two stimuli names to either side of an equal sign (the relational operator; e.g., *A* = *B*).
Step 1: Locate relational operator and assign to a variable (*r*).
Step 2: Assign all characters to the left of (*r*) to a variable (*x*).
Step 3: Assign all characters to the right of (*r*) to a variable (*y*).
Step 4: Return derived relational statement arranged (*y*)(*r*)(*x*).
Output: One relational statement with stimuli names reflected across the equals sign (e.g., *B* = *A*).

#### Combinatorial Entailment

At the level of combinatorial entailment, equivalence relations are defined by what Sidman called transitivity. That is, the combination of two relational statements of equivalence that each involved one shared stimulus (e.g., *A* = *B* and *B* = *C*) resulted in equivalence responses to the other two stimuli (e.g., *A* = *C*). This transitivity of equivalence is experienced when the word of something previously known is learned in another language by being told that the new word is the same as the old word. Combinatorial entailment for combining an equivalence relation with other non-equivalence relations will be covered later so the pseudo code for combinatorial entailment of two equivalence relations would look something like the following:

Input: Two relational statements each composed of two stimuli names to either side of an equals sign (e.g., *A* = *B* and *B* = *C*).
Step One: Assign each relational statement to their own variable (*a* & *b*).
Step Two: Locate the relational operator in (*a*) and assign to variable (*ra*).
Step Three: Assign all characters to the left of (*ra*) to a variable (*xa*).
Step Four: Assign all characters to the right of (*ra*) to a variable (*ya*).
Step Five: Locate the relational operator in (*b*) and assign to variable (*rb*).
Step Six: Assign all characters to the left of (*rb*) to a variable (*xb*).
Step Seven: Assign all characters to the right of (*rb*) to a variable (*yb*).
Step Eight: Count the number of unique stimuli names in the list (*xa*, *xb*, *ya*, *yb*) and assign to a variable (*u*).
Step Nine: Count the number of items in the list (*xa*, *xb*, *ya*, *yb*) and assign to a variable (*c*).
Step Nine: IF (*u*) is equal to (*c*) minus 1, continue, ELSE STOP.
Step Ten: Find the stimuli names in the list (*xa*, *xb*, *ya*, *yb*), that only occur once and assign to variables (*da*) and (*db*).
Step Eleven: Return derived relational statement arranged (*da*)(*ra*)(*db*).
Output: One relational statement with stimuli names of the unique stimuli on either side of the equals sign (e.g., *A* = *C*).

### Greater Than and Less Than Derivation

#### Mutual Entailment

All relational responses other than equivalence do not demonstrate symmetrical responding at the level of mutual entailment (Hayes et al., 2001, pp. 29–31). In the case of the comparative relational pair Greater Than (>) and Less Than (<), the mutually derived relational response of one is the other. For example, the mutually entailed relationship to “A dime is greater than a nickel” is “A nickel is less than a dime.” Pseudo code for mutual derivation of these two relations would look something like the following:

Input: One relational statement composed of two stimuli names to either side of a left carrot (<) or right carrot (>) sign. (e.g., *A* < *B*).
Step One: Locate the relational operator and assign to variable (*r*).
Step Two: Assign all characters to the left of (*r*) to a variable (*x*).
Step Three: Assign all characters to the right of (*r*) to a variable (*y*).
Step Four: When (*r*) is (<), assign (>) to variable (*rm*), else assign (<) to variable (*rm*).
Step Five: Return derived relational statement arranged (*y*)(*rm*)(*x*).
Output: One relational statement with stimuli names reflected across the relational operator and the relational operator swapped with its mutually entailed alternative. (e.g., *B* > *A*).

#### Combinatorial Entailment

At the level of combinatorial entailment, comparative relations do not demonstrate transitivity as described by Sidman. That is, just because at least one of the two relational statements includes a comparative does not necessarily determine that the combinatorially entailed relation will be the same. For example, the derived relation between unique stimuli when combining the statements “A lemon is less than a potato” and “An apple is less than a lemon” is “A potato is greater than an apple.” Additionally, special cases of combinatorial entailment with comparative relations are logically indeterminate (Vitale et al., 2008, 2012; Quinones and Hayes, 2014). In the same example above, if the second statement had been “An apple is greater than a lemon,” there would not be enough information provided to fully derive a specific comparative relation between the potato and apple. That is, given the available information it is equally possible that the potato and apple are equal or different for the characteristic referenced by the comparative relation. The following pseudo code should handle all cases of combinations of at least one greater than or less than relational statement and a second greater than or less than or an equivalence statement and return a derivable relation where possible and some indication when an indeterminate case occurs.

Input: Two relational statements each composed of two stimuli names to either side of a relational operator with at least one being Greater Than (>) or Less Than (<; e.g., *A* < *B* & *B* < *C*).
Step One: Assign each relational statement to their own variable (*a* & *b*).
Step Two: Locate the relational operator in (*a*) and assign to variable (*ra*).
Step Three: Assign all characters to the left of (*ra*) to a variable (*xa*).
Step Four: Assign all characters to the right of (*ra*) to a variable (*ya*).
Step Five: Locate the relational operator in (*b*) and assign to variable (*rb*).
Step Six: Assign all characters to the left of (*rb*) to a variable (*xb*).
Step Seven: Assign all characters to the right of (*rb*) to a variable (*yb*).
Step Eight: Count the number of unique stimuli names in the list (*xa*, *xb*, *ya*, *yb*) and assign to a variable (*u*).
Step Nine: Count the number of items in the list (*xa*, *xb*, *ya*, *yb*) and assign to a variable (*c*).
Step Nine: IF (*u*) is equal to (*c*) minus 1, continue, ELSE STOP.
Step Ten: Find the stimuli names in the list (*xa*, *xb*, *ya*, *yb*), that only occur once and assign to variables (*da*) and (*db*).
Step Eleven (see “Sequence Matters”): Based on sequence of (*da* & *db*) in (*a* & *b*) and the combination of (*ra* & *rb*) assign (<), (>), or (indeterminate) to (*ra*).
Step Twelve: Return derived relational statement arranged (*da*)(*ra*)(*db*).
Output: One relational statement with stimuli names of the unique stimuli on either side of the appropriate relational operator OR “indeterminate” in cases where the relation cannot be derived from the input statements (e.g., *A* < *C*).

## Results/Tutorials

The most current *R* script files may be downloaded.^1^ The two scripts are task specific. The first script (relational permutation. *R*), intakes the relational base set and outputs the derived relational set list. The second script (network graph visualizer. *R*), intakes the output from the first and outputs network graph visualizations. This separation was done so that the derivation processes may be deployed in standalone applications without the additional overhead of the visualization dependencies. The following tutorial walks through how to generate solution sets for the two most common three stimulus relational networks discussed in RFT literature.

### Tutorial 1: Three Stimulus Equivalence Network

A stimulus equivalence network is any set of stimulus where the relationship between each is equivalence (i.e., sameness). Many RFT publications use the example of an equivalence network connecting the letters of a word, the sound, and the referent object as an introduction to the stereotypical three stimulus network. For example, the letters D-O-G are a distinct stimulus from the sound “dog” and the animal that the English language refers to as a dog and yet once learned, most individuals will respond to all three functionally the same. In this package, each stimulus is represented by a character (i.e., 0–9, *a*–*z*, or *A*–*Z*) and the relation is represented by a relational operator (i.e., =, <, >, and “ku”). In this example, the stimuli letters: DOG, sound: “dog,” and object: dog will be represented by A, B, and C, respectively. The equivalence relation between each will be represented by the equals sign (=). The simplest set of relational statements that derives to the entire equivalence network are any two statements that involve all three stimuli. Those may take the form “A = B, B = C,” “C = A, B = A,” or any other of the possible permutations afforded by the bi-directional nature of the equivalence relation. For purposes of this tutorial, “A = B” and “B = C” will be the base set.

Step One: Open the “relational permutation. R” script, load dependent libraries and the “relationParse,” “mutualEntail,” “combinatorialEntail,” and “relationTrain” functions.
Step Two: Assign base set to a list with a variable name (*a*).
c(“A=B”,“B=C”) -> a
  Notes:
  1. This example uses “a” as a generic variable name but any one can be used as long as you are consistent throughout the code.
  2. The left to right syntax used here is for sequential readability by R coding novices. R handles a number of different syntax structures. In pseudo code this line would read as “Combine (c(…)) the character strings “A = B” and “B=C” into a vector list and assign (−>) to the variable named “a.”
Step Three: Assign the output of the relational derivation function with (*a*) as the input to a variable (*x*).
relationTrain(a) -> x
  Notes: The relationTrain output is a table of all the base and derived relational statements.It includes additional information used for visualization and diagnostic purposes. The table (*x*) should look like Table 1.
  **Table 1:** RelationTrain output for three stimulus equivalence network.

From	To Relation_Type	Derivation_Level	Relation	Edge_color	Derived_from
A	*B* =	Directly Trained	*A = B*	Blue	NA
B	*A* =	Mutually Entailed	*B = A*	Purple	NA
B	*C* =	Directly Trained	*B = C*	Blue	NA
C	*B* =	Mutually Entailed	*C = B*	Purple	NA
A	*C* =	Combinatorially Entailed	*A = C*	Orange	*A = B,B = C*
C	*A* =	Combinatorially Mutually Entailed	*C = A*	Orange	*A = B,B = C*
Step Four: Open the ‘network graph visualizer. *R*’ script and load dependent libraries.
Step Five: Using (*x*), create a list of unique relational statements and assign to variable (edgeList).
filter(x,!duplicated(Relation))-> edgeList
  Note: While there is a constrained set of derivable relationships, relation Train can sometimes create duplicates since a relation may be derived *via* both combinatorial and mutual entailments.
Step Six: Coerce (edgeList) into a network object type and assign to a variable (relTnet).
network(edgeList, matrix.type='edgelist',  ignore.eval=FALSE, loops=TRUE,  directed = FALSE, multiple = TRUE)->  relTnet
Step Seven: Coerce (relTnet) into an Igraph object and assign to variable (rel_graph).
asIgraph(relTnet) -> rel_graph
Step Eight: Plot (rel_graph) to generate the network graph visualization.
plot(rel_graph,  vertex.size=8, vertex.label=V(rel_graph)$vertex.names, edge.label=E(rel_graph)$Relation, edge.color=E(rel_graph)$edge_color, edge.arrow.size=1)
  Note: Output plot should match Figure 1.
  **Figure 1:**
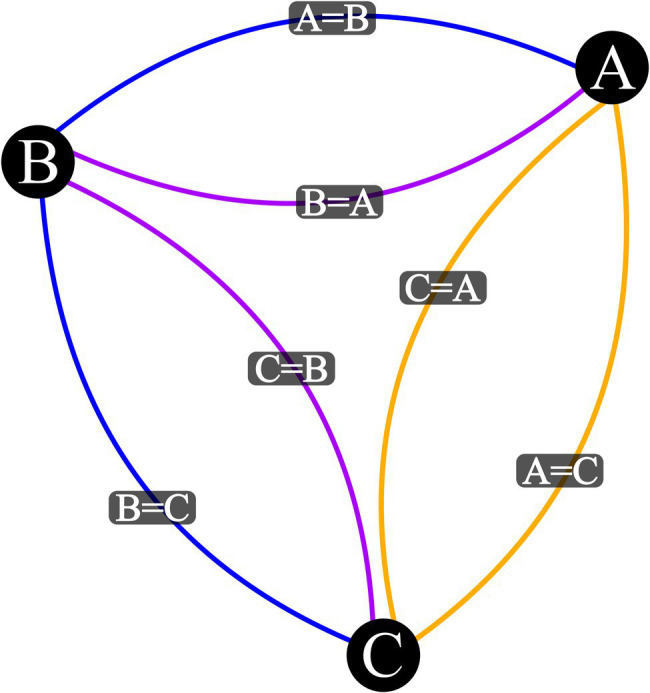
Visualization Output for three Stimulus Equivalence Network with a Base Relational Set of *A* = *B* and *B* = *C*. Vertex represent stimuli, blue edges are input relations, purple edges are mutually derived relations, and orange edges are combinatorially derived relations.
  Notes:
  1. Steps four through eight are already written in the ‘network graph visualizer. *R*’ script so they only need to be executed. They are spelled out here for illustration purposes.
  2. Code includes additional line by line commentary to signpost how various settings affect visualization output.

### Tutorial 2: Three Stimulus Mixed Relations Network

Very little changes in terms of coding between an equivalence network and one that includes either all comparative relations or mixed relations. Below will only cover those changes. Steps that are identical to Tutorial 1 will be abbreviated and indicated with ellipses (…). For tutorial purposes, the base set used will be “A > B” and “B=C.”

Step One: Open the relational permutations. *R* script…
Step Two: Assign base set to a list with a variable name (*a*).
c("A>B","B=C") -> a
Step Three: Assign output to a variable …
  Note: The table generated by Step Three (*x*) should look like Table 2.
  **Table 2:** RelationTrain output for three stimulus mixed relation network.

From	To Relation_Type	Derivation_Level	Relation	Edge_color	Derived_from
A	*B* >	Directly Trained	*A > B*	Blue	NA
B	*A* <	Mutually Entailed	*B < A*	Purple	NA
B	*C* =	Directly Trained	*B = C*	Blue	NA
C	*B* =	Mutually Entailed	C=B	Purple	NA
A	*C* >	Combinatorially Entailed	*A > C*	Orange	*A > B, B = C*
C	*A* <	Combinatorially Mutually Entailed	*C < A*	Orange	*A > B, B = C*
Step Four: Open network graph visualizer. *R* script…
Step Five: Create an edge list…
Step Six: Coerce edge list to network object…
Step Seven: Coerce network object to an igraph object…
Step Eight: Plot Network Visualization…
Note: Output plot of Step Eight should match Figure 2.

**Figure 2:**
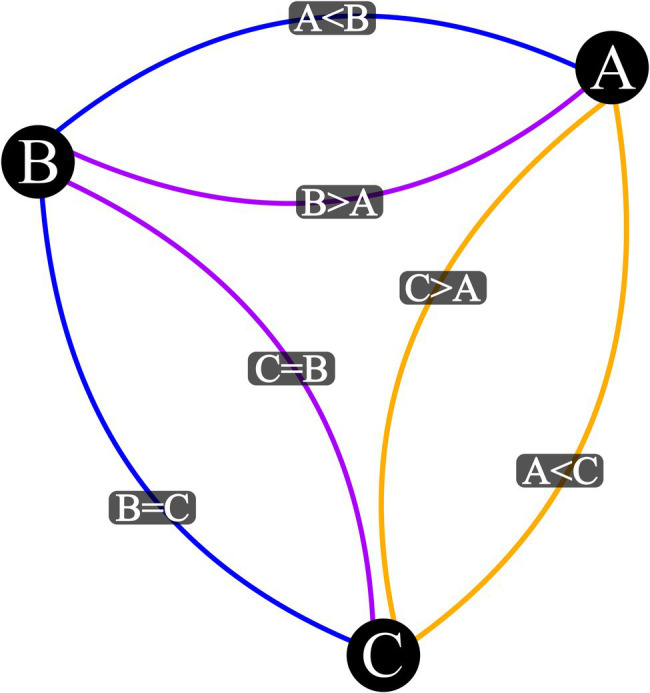
Visualization Output for three Stimulus Mixed Relation Network with a Base Relational Set of *A* > *B* and *B* = *C*. Vertex represent stimuli, blue edges are input relations, purple edges are mutually derived relations, and orange edges are combinatorially derived relations.

### Tutorial 3: Complex Mixed Relations Network

While the stereotypical two base relation (three stimulus) networks are used for the first two tutorials, the package is not limited to only two base relation sets. It can handle single relation inputs (i.e., “*A* > *B*”) as well as many relations as the base set input. For this example, the base set will be “*gkuj*,” “*h* < *i*,” “*i* < *k*,” “*k* < *j*,” “*l* < *o*,” “*mkuk*,” “*n* = o,” and “*p* > o” to highlight how little changes in the actual code.

Step One: Open the relational permutations. *R* script…
Step Two: Assign base set to a list with a variable name (*a*).
c("gkuj", "h<i", "i<k", "k<j", "l<o", "mkuk", "n=o", "p>o") -> a
Step Three: Assign output to a variable …
  Note:
  The table generated by Step Three (*x*) should look like Table 3.
  **Table 3:** RelationTrain output for complex mixed relation network.

From	ToRelation_Type	Derivation_Level	Relation	Edge_color	Derived_from
*g*	*jku*	Directly Trained	*gkuj*	Blue	NA
*h*	*i<*	Directly Trained	*h < i*	Blue	NA
*i*	*h>*	Mutually Entailed	*i > h*	Purple	NA
*i*	*k<*	Directly Trained	*i < k*	Blue	NA
*j*	*gku*	Mutually Entailed	*jkug*	Purple	NA
*j*	*k>*	Mutually Entailed	*j > k*	Purple	NA
*k*	*i>*	Mutually Entailed	*k > i*	Purple	NA
*k*	*j<*	Directly Trained	*k < j*	Blue	NA
*k*	*mku*	Mutually Entailed	*kkum*	Purple	NA
*l*	*o<*	Directly Trained	*l < o*	Blue	NA
*m*	*kku*	Directly Trained	*mkuk*	Blue	NA
*n*	*o=*	Directly Trained	*n = o*	Blue	NA
*o*	*l>*	Mutually Entailed	*o > l*	Purple	NA
*o*	*n=*	Mutually Entailed	*o = n*	Purple	NA
*o*	*p<*	Mutually Entailed	*o < p*	Purple	NA
*p*	*o>*	Directly Trained	*p > o*	Blue	NA
*h*	*k<*	Combinatorially Entailed	*h < k*	Orange	*h* < *i*, *i* < *k*
*i*	*j<*	Combinatorially Entailed	*i < j*	Orange	*i* < *k*, *k* < *j*
*j*	*i>*	Combinatorially Mutually Entailed	*j > i*	Orange	*i* < *k*, *k* < *j*
*k*	*h>*	Combinatorially Mutually Entailed	*k > h*	Orange	*h* < *i*, *i* < *k*
*l*	*n<*	Combinatorially Entailed	*l < n*	Orange	*l* < *o*, *n* = *o*
*l*	*p<*	Combinatorially Entailed	*l < p*	Orange	*l* < *o*, *p* > *o*
*n*	*l>*	Combinatorially Mutually Entailed	*n > l*	Orange	*l* < *o*, *n* = *o*
*n*	*p<*	Combinatorially Entailed	*n < p*	Orange	*n* = *o*, *p* > *o*
*p*	*l>*	Combinatorially Mutually Entailed	*p > l*	Orange	*l* < *o*, *p* > *o*
*p*	*n>*	Combinatorially Mutually Entailed	*p > n*	Orange	*n* = *o*, *p* > *o*
*h*	*iku*	Combinatorially Mutually Entailed	*hkui*	Orange	*i* < *k*, *h* < *k*
*h*	*j<*	Combinatorially Entailed	*h < j*	Orange	*h* < *i*, *i* < *j*
*i*	*hku*	Combinatorially Entailed	*ikuh*	Orange	*i* < *k*, *h* < *k*
*i*	*kku*	Combinatorially Entailed	*ikuk*	Orange	*h* < *i*, *h* < *k*
*j*	*h>*	Combinatorially Entailed	*j > h*	Orange	*k* < *j*, *h* < *k*
*j*	*kku*	Combinatorially Mutually Entailed	*jkuk*	Orange	*i* < *k*, *i* < *j*
*k*	*iku*	Combinatorially Entailed	*kkui*	Orange	*k* < *j*, *i* < *j*
*k*	*jku*	Combinatorially Entailed	*kkuj*	Orange	*i* < *k*, *i* < *j*
*l*	*nku*	Combinatorially Entailed	*lkun*	Orange	*l* < *p*, *n* < *p*
*l*	*oku*	Combinatorially Mutually Entailed	*lkuo*	Orange	*p* > *o*, *l* < *p*
*n*	*lku*	Combinatorially Mutually Entailed	*nkul*	Orange	*l* < *p*, *n* < *p*
*n*	*oku*	Combinatorially Mutually Entailed	*nkuo*	Orange	*l* < *o*, *l* < *n*
*n*	*pku*	Combinatorially Entailed	*nkup*	Orange	*l* < *n*, *l* < *p*
*o*	*lku*	Combinatorially Entailed	*okul*	Orange	*p* > *o*, *l* < *p*
*o*	*nku*	Combinatorially Entailed	*okun*	Orange	*l* < *o*, *l* < *n*
*o*	*pku*	Combinatorially Entailed	*okup*	Orange	*l* < *o*, *l* < *p*
*p*	*nku*	Combinatorially Mutually Entailed	*pkun*	Orange	*l* < *n*, *l* < *p*
*p*	*oku*	Combinatorially Mutually Entailed	*pkuo*	Orange	*l* < *o*, *l* < *p*
*h*	*kku*	Combinatorially Mutually Entailed	*hkuk*	Orange	*k* < *j*, *h* < *j*
*i*	*jku*	Combinatorially Entailed	*ikuj*	Orange	*h* < *i*, *h* < *j*
*j*	*iku*	Combinatorially Mutually Entailed	*jkui*	Orange	*h* < *i*, *h* < *j*
*k*	*hku*	Combinatorially Entailed	*kkuh*	Orange	*k* < *j*, *h* < *j*
Step Four: Open network graph visualizer. *R* script …
Step Five: Create an edge list …
Step Six: Coerce edge list to network object …
Step Seven: Coerce network object to an igraph object …
Step Eight: Plot Network Visualization …
  Note:
  Output plot of Step Eight should match Figure 3.

**Figure 3:**
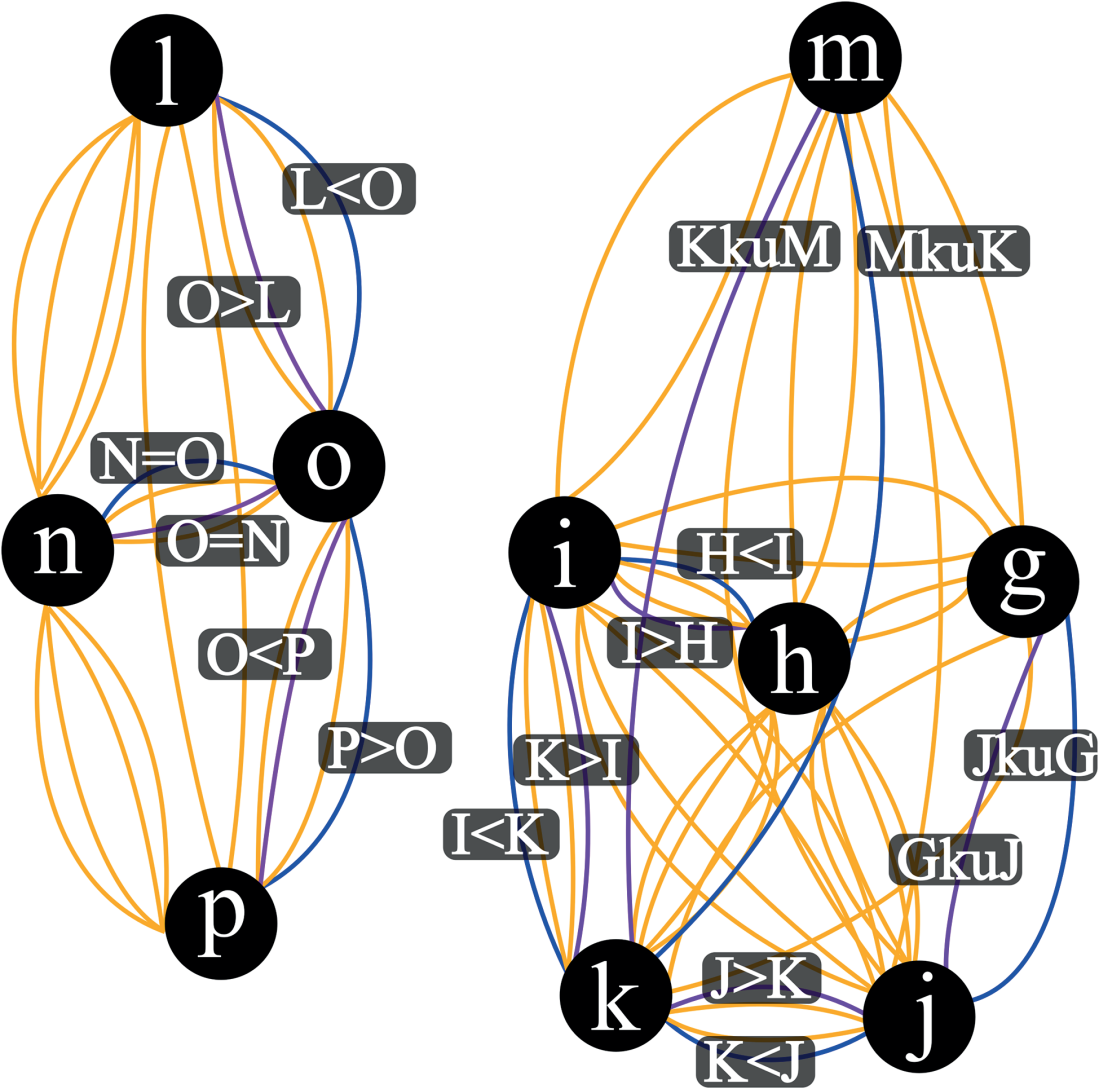
Visualization Output for Complex Mixed Relation Network. This also illustrates how networks may be isolated from one another. Vertex represent stimuli, blue edges are input relations, purple edges are mutually derived relations, and orange edges are combinatorially derived relations. ^*^Combinatorially derived relational labels omitted for publication readability.

## Discussion

### Sequence Matters

In the process of converting the various metaphors of comparative relational derivation behavior into computer code logic it became apparent that the deeper implication of intransitivity is that the input sequence of relational statements impacts how the code derives output. Vitale et al. (2008, 2012) highlighted related sequential effects and identified six major sequence forms when two greater than and less than statements are combined. These forms are based on a combination of the similarity of the two relations and the linearity of the sequence of stimuli presented. For example, a simple relational pair *A* > *B* & *B* > *C* is described as having the same relational operator and presenting linearly. In contrast, *A* > *B* & *C* < *A* present mixed relational operators and a non-linear arrangement of statements. This method of structurally parsing comparative relational pairs identified 48 unique forms of possible inputs when constrained to only comparative pairs. Allowing for one statement of the input pair to be an equivalence statement expands the possible unique input forms to 72 unique forms falling into seven major sequence forms.

The task required in step 11 above requires a conditional match to one of those seven major forms to identify the output relational operator. In the provided R package, this was accomplished by a series of nested conditional IF ELSE statements that first identify the shared stimuli and their relative locations and then compares the relational operators and works through a matching tree until a final TRUE value returns the appropriate (*ra*) relational operator value. Alternatively, this step 11 task may be accomplished through a lookup table, but in either method, the input sequence of each relational statement cannot be ignored or discarded.

### Two Input, One Output

The pseudo code presented above for each combinatorial entailment variant outputs one relational statement. According to RFT, two relational statements are a product of combinatorial entailment. This seeming contradiction is not actually a shortcoming of the code or theory. In the metaphor of derivation provided by the theory, either relational statement between unique stimuli may be derived combinatorially. In implementation, however, to use the combinatorial entailment script to derive both statements would require more computation than to feed the single output of the combinatorial entailment script right into the input of the mutual entailment script. This nesting of combinatorial and mutual entailment scripts yields a savings of ~ 5–7 steps or ~ 50–64%.While this may not be the exact method in which relational derivation occurs in human organisms, the exponential expansion of relational networks implies that the computational efficiency gain here is increasingly significant as input sets grow in size and complexity.

### Indeterminate but Known Derived Relations

In the special case of comparative relations not providing enough information to explicitly derive a comparative or equivalence relationship there is a pragmatic issue of how to handle these exceptions computationally. The two immediate possibilities are to (1) treat these cases as errors and not include them in any further derivation or the final output, or (2) treat these cases as any other derivable relation but with their own special properties that impact mutual and combinatorial derivation processes. The first option is reasonable but may contradict both RFT and expectations of human behavior. The full discussion of this contradiction is not within the scope of this paper and will be described in more detail in related publications. The short explanation is that individuals engaging in derivation can respond with respect to a derived relation between two stimuli without knowing more about that relationship. Consider the example of missing puzzle pieces. An individual presented with two pieces of a puzzle that do not fit together will engage in responses that reflect the absence of one or more pieces with only the partial knowledge of the shape and colors of the present pieces. Similarly, when provided two relational statements that describe metaphorically neighboring stimuli (e.g., “Neveah is taller than Damerus,” and “Meara is taller than Damerus.”) individuals will respond to the unique stimuli as if they were related to each other in some way.

An additional paradox of responding to a relation that cannot be derived exactly from information presented is that many times the response will be accurate and consequently reinforced as if the behaving individual had known the appropriate relation all along. For these reasons, the provided R package has been implemented using the second option outlined in this section. Additionally, to maintain naming consistency with the upcoming publication detailing this unique relational response, instead of “indeterminate,” the package outputs “ku” (an abbreviation of “Known-Unknown”) as the relational operator for those special case derived relations that cannot be otherwise resolved to equivalence, greater than, or less than. Additionally, KU relations are handled as expressing a dominant transitivity in all mutual and combinatorial derivations. That is, a KU relation will mutually entail another KU relation and any relational pair that includes at least one KU relation will combinatorially derive to a KU relation.

### Relational Recursion

The provided R package was designed to derive all possible relations implied by a base set. In simple one, and few, relational statement base sets, the complete derivable set is within one mutual and combinatorial derivation cycle away from the base set. This is not the case as the size of the base set increases. For that reason, the package will iteratively recombine the base and derived relational statements until all non-KU relational statements have been derived. The package handles this process in two step fashion. First, all pair sets input into the combinatorial entailment script are validated as being unique to each other (not two of the same statement) and not being both KU statements. This prevents logic loops where the script just continuously takes in functionally identical statements expressed in both unique sequences (i.e., *A* < *B* & *B* > *A*) and outputs the same as well as the infinite derivation that occurs when both statements are KU relations. Second, the final output relational statement list of each derivation iteration is compared against the most recent full relational statement list prior to that iteration and as long the output exceeds input, the two lists are merged by unique relational statements and the merged list becomes the input for the next iteration of derivation. Once an output list has zero unique relations statements compared to the input list, the recursion process ends and the final solution set is output to a table as well as input into the visualization script that generates the network graph visualization.

### Limitations

This package has several limitations that fall into philosophical and technical categories. Philosophically, it would be a critical scientific error to assume that humans are perfect deriving logic machines or that the output of this package applies independent of context. Technically, this package is a prototype and should be used with a general approach of *caveat emptor* with specific awareness of the limitations that come with it being built on a small relational set, requiring specific and limited input forms, assuming singular context for all input relations, and having limited evaluation methods.

#### Humans Are Not Logic Machines

The pragmatic goal of this software package is to open possibilities in empirical research and application of RFT related work and for relational reasoning research more generally. It is also meant to provide a learning tool for what is likely to become available as development of relational computer models continues. The method that has been implemented here requires translating theory about human behavior into a repeatable logical process a computer can execute. That means that the output is, at best, an approximation of what may be expected in human behavior within the constraints of declaratory logic required by computers. This output may be reliable and may reasonably approximate human behavior under many common conditions but conflating human behavior and computer logic is a mistake in both research and application. An example is provided by the known-unknown relational response discussed above. In all of the available basic research on known-unknowns, human participants reliably did not respond accurately to derived relations that were lacking sufficient information (Saunders et al., 1988; Vitale et al., 2008, 2012; Quinones and Hayes, 2014). Simply put, human behavior was not strictly logical, and a variety of conditions may contribute to unexpected responses when the software accurately derives logically consistent relationships such as known-unknowns. For this reason, the software intentionally does not check for contradictory relationships. A base set input list such as “*A* > *B*,” “*B* = *C*,” & “*C* > *A*” will be handled the same as any other input list. In this case, the package will output that the relations “*A* < *C*” and “*A* > *C*” which are both logically derivable within the larger solution set despite also being logically paradoxical.

#### Context Matters

A keystone characteristic of RFT is contextual control. That is, human behavior is specific to contextual environments. In other words, a behavior that occurs reliably following a specific stimulus in one context, may never occur following the same stimulus in another context. Additionally, an opposing functional response may occur reliably in that second context in the presence of the same stimulation. RFT delineates two categories of stimulus influence named Crel and Cfunc (pronounced see-rell and see-funk), the latter of which is described as selecting for the contextual function most likely to be reinforced. This package, as a first attempt, focused on the simpler task of deriving Crels (contextual relations) and assuming a singular Cfunc. If a user wishes to model multiple context dependent relational sets, the base set for each context should be run in independent instances at this time.

#### Limited Relational Set

As a prototype, the relational set modeled was significantly reduced from what has been described in the RFT literature. The goal of modeling equivalence, greater than, and less than as the prototype set of relations resulted in either modeling for the known-unknown cases or ignoring an interesting relational phenomenon. Even amongst the comparative relational response family, this constrained set excluded relational types such as Difference and Opposition. Future goals of this package include developing such derivation logic for each of the relational response class families, but for the moment, the package is similarly constrained.

#### Limited Input Structure

Related to the strategic choice to simplify above, the package is also subjectively constrained to a specific input form for now. Relational statements used as input must follow a “one character—relational operator—one character” format. This format was adopted because much of the existing RFT literature uses a letter or numeral as convention to designate individual stimuli (e.g., Lemon = A or Dog = B). In this way, stimulus pairs and their relation can be written in a shorthand such as A! = B (i.e., “Lemons are different from dogs”).This shorthand convention has been used throughout this paper and became the prototypical input structure. Other RFT researchers have proposed a more rigorous relational symbolic technical notation (McLoughlin et al., 2019) as an elaboration of the notation used since the earliest days of RFT (Hayes, 1991), but this notation has not been widely adopted by the field yet and requires characters not easily input using a standard QWERTY keyboard. While such rigorous notation may be the future of the field and may be integrated into future releases of this package, the current release reliably handles the relational operands equals (=), less than (<), greater than (>), and known-unknown (ku). This leaves all ten numerals (0–9) and 26 alphabetic symbols to represent a single stimulus. Additionally, alphabetic characters are case sensitive in the package so “A” is handled as distinct from “a” so there are enough input permutations to represent a very extensive relational base set. At this time, non-alphanumeric characters (e.g., #, $, %, etc.) have not been tested in the input so it may be best practice to avoid such symbols, or at least use them with caution.

#### Limited Evaluation Methods

While graph theory provides many evaluation methods for structural assessment of network graphs as well as relational database languages (e.g., NEO4J), there are no currently known systematized network evaluation methods that apply to relational derivation output as described in this manuscript. In lieu of that, stereotypical examples of trained and derived sets previously published in RFT work, as well as sets generated and verified by hand by the first author (PS), were used as assessment tools to validate the output of the software. For example, Vitale et al. (2008, p. 369) provides 48 unique arrangements of comparative pairs of base relational statements (e.g., A > B; B > C) that they had previously manually solved for the mutually and combinatorially derived relations. Each of these base pairs were run through the package and the output checked against the original key for agreement. There was zero disagreement in this comparison set. Similar equivalence and mixed relations base sets were generated during development to compare the package output against expectations. Several were left in the code, with clarifying comments, for users to run themselves as additional demonstrations. In all cases, if output did not match expectations, manually generated keys were double checked for accuracy and code was inspected to diagnose the disagreements.

This means that any errors in the validation data may have resulted in errors in the coded processes. Until wider adoption and systematic validation iron out bugs, output should be generally checked against the user’s expectations. In the future more robust validation tools that do not rely on iterative permutation of base relational sets may need to be developed to deal with highly complex networks.

### Looking Forward

The initial development of this package was largely motivated by the number of human hours it took the first author (PS) to manually derive networks when planning RFT research or reading published experiments. If this package relieves even one other person of that task (and the frustration of finding an incorrectly derived relation in their handwritten notes) then it will have been worth the effort. Beyond the immediate benefit of saving labor, it is hoped that this package will pave another step in the bridge between basic research procedures and human complexity. It will only be when the basic account of behavior can be demonstrated convincingly, using reasonably complex and yet still interpretable models, that the exposure to an extrapolation critique will be minimized. By providing a reliable, repeatable, and human readable method of generating more complex relational models, we hope that procedural complexity may become more accessible in research and practice. Along these same lines, this package may be used to survey the current relational experimental and applied literature procedures. The process of converting behavioral phenomena to abstract relational representations and then deriving their implications and integrating that into a procedural description is tedious and prone to human error. If applied systematically to published procedures, this tool may help clarify where conflicting data may be due to such sources of confusion.

One insight into relational network models that only came to be obvious once graphed was that of multiple isolated networks as illustrated in Figure 3. In this example, when the base relational set was written in text as “*gkuj*,” “*h* < *i*,” “*i* < *k*,” “*k* < *j*,” “*l* < *o*,” “*mkuk*,” “*n* = *o*,” and “*p* > *o*,” there is no indication that this string of characters will be arranged such that there are no relations between the stimulus sets of“*l*, *n*, *o*, *p*” and “*g*, *h*, *i*, *j*, *k*, *m*.” This isolation of these two networks is very clear in graph form but it is not immediately noticeable even when viewed as a derived tabular list, such as in Table 3. This isolation is not limited to that one specific base set. There are a number of additional large base sets provided in the code of the relational permutations. R code that can be run to see three and four isolated network examples. If a practitioner were to go to the effort of parsing a client dialogue into stated relations a la Belisle et al. (2018) and then input those as a base set into this package, isolation of networks as we see in this example may provide immediate and actionable insight into key relational connections that a client may not be currently responding to and may vastly alter derivations across the rest of their presenting network. While this implication is far from being tested at the moment, this may provide a pathway toward a client focused functional analysis of complex human language.

Relational learning presents an experimental and conceptual challenge because of its extreme expansivity, and with it the possibility of conceptual confusion and error. This software package is designed to help address those limitations. By prototyping relational network graph visualizations consistent with RFT and other approaches to relational learning users can better prepare for evaluation and collaboration with computer analog systems such as blended DNN and SOM architectures that are currently being developed.

Future opportunities for development that are directly related to the package include:

- publication on CRAN and an associated “Shiny” web app to reduce technical barriers to adoption of the package,
- developing novel methods of validating derivation processes,
- integrating edge weighting as outlined in Edwards et al. (2022),
- expanding the relational classes handled by the package,
- integrating context characteristics for discriminating between relations occurring under specific context,
- automating key parts of RFT research and training development processes, and
- and integration with Natural Language Processing algorithms similar to how word2vec (Mikolov et al., 2013) has been applied to early dementia detection (Rutkowski et al., 2021).

## Additional Information

Project link: https://github.com/CoachPatrickSmith/Relational-Network-Generator.git.

Operating System: Platform Independent.

Programming Language: R Statistical Software.

Restrictions on Non-academic Usage: GPLv3 license.

## Data Availability Statement

The datasets presented in this study can be found in online repositories. The names of the repository/repositories and accession number(s) can be found at: https://github.com/CoachPatrickSmith/Relational-Network-Generator.

## Author Contributions

PS authored the R script and wrote the original manuscript draft. SH provided major revisions to the Introduction and Discussion sections, minor revisions to all other sections, and advised throughout the entire manuscript and script development process. All authors contributed to the article and approved the submitted version.

## Conflict of Interest

The authors declare that the research was conducted in the absence of any commercial or financial relationships that could be construed as a potential conflict of interest.

## Publisher’s Note

All claims expressed in this article are solely those of the authors and do not necessarily represent those of their affiliated organizations, or those of the publisher, the editors and the reviewers. Any product that may be evaluated in this article, or claim that may be made by its manufacturer, is not guaranteed or endorsed by the publisher.

## Footnotes

^1^ https://github.com/CoachPatrickSmith/Relational-Network-Generator.git
